# Previously described sequence variant in *CDK5RAP2 *gene in a Pakistani family with autosomal recessive primary microcephaly

**DOI:** 10.1186/1471-2350-8-58

**Published:** 2007-09-01

**Authors:** Muhammad Jawad Hassan, Maryam Khurshid, Zahid Azeem, Peter John, Ghazanfar Ali, Muhammad Salman Chishti, Wasim Ahmad

**Affiliations:** 1 Department of Biochemistry, Faculty of Biological Sciences, Quaid-i-Azam University, Islamabad, Pakistan

## Abstract

**Background:**

Autosomal Recessive Primary Microcephaly (MCPH) is a disorder of neurogenic mitosis. MCPH leads to reduced cerebral cortical volume and hence, reduced head circumference associated with mental retardation of variable degree. Genetic heterogeneity is well documented in patients with MCPH with six loci known, while pathogenic sequence variants in four respective genes have been identified so far. Mutations in *CDK5RAP2 *gene at MCPH3 locus have been least involved in causing MCPH phenotype.

**Methods:**

All coding exons and exon/intron splice junctions of *CDK5RAP2 *gene were sequenced in affected and normal individuals of Pakistani MCPH family of Kashmiri origin, which showed linkage to MCPH3 locus on chromosome 9q33.2.

**Results:**

A previously described nonsense mutation [243 T>A (S81X)] in exon 4 of *CDK5RAP2 *gene has been identified in the Pakistani family, presented here, with MCPH Phenotype. Genomic and cDNA sequence comparison revealed that the exact nomenclature for this mutation is 246 T>A (Y82X).

**Conclusion:**

Recurrent observation of Y82X mutation in *CDK5RAP2 *gene in this Pakistani family may be a sign of confinement of a rare ancestral haplotype carrying this pathogenic variant within Northern Pakistani population, as this has not been reported in any other population.

## Background

Autosomal recessive primary microcephaly (MCPH), a rare neurodevelopmental disorder, is characterized by reduced head circumference of at least 4 standard deviations below the age- and sex- related population specific means; associated with non progressive mental retardation of variable degree but no other neurological deficit. In individuals with MCPH phenotype, the brain weight is markedly reduced and the cerebral cortex is disproportionately small with no major abnormalities in cortical architecture [1,2]. MCPH is genetically heterogeneous with six loci (MCPH1–MCPH6) mapped to date [3-8] while four of the corresponding genes [Microcephalin (MIM-607117) at MCPH1, *CDK5RAP2 *(MIM-608201) at MCPH3, *ASPM *(MIM-605481) at MCPH5 and *CENPJ *(MIM-609279) at MCPH6] have been identified [9-11].

The MCPH5 is most prevalent in patients with primary microcephaly world wide and at least 30 pathogenic sequence variants in the corresponding gene *ASPM *(abnormal spindle like microcephaly associated) are known to date without any significant correlation between position of mutation and severity of microcephaly [12-16]. *ASPM *has preferential expression in neuroepithelium of the lateral ventricles during neurogenic cycle and its various isoforms with different number of IQ motifs have been detected in numerous tissues [17]. Four mutations have been reported in Microcephalin gene at MCPH1 locus, causing MCPH and an allelic form, PCC (Premature chromosome condensation) syndrome (PCC; 606858), characterized by microcephaly, short stature, and misregulated chromosome condensation [9,18-20]. Microcephalin functions as a proximal factor in the DNA damage checkpoints that control multiple damage sensors and early mediators and also implicated in cell cycle checkpoints, controlling and regulating other important molecules and thus affecting the timing of mitosis; its depletion abolishes the DNA damage response and results in centrosomal abnormalities and chromosomal aberrations [21-23]. Three mutations in the *CENPJ *(centromere associated protein J) gene at MCPH6 locus are known in Brazilian and Pakistani families with autosomal recessive primary microcephaly [11,24]. *CENPJ *encodes a centrosomal protein which is associated with γ-tubulin ring complex and in-vitro evidence suggested its role in inhibition of microtubule nucleation [25]. Therefore, a role for *CENPJ *in centrosome duplication at the beginning of mitosis has been proposed [26].

Mutations in Cyclin-dependent protein kinase 5, regulatory subunit associated protein 2, (*CDK5RAP2*) gene at MCPH3 locus are least involved in causing MCPH phenotype. To date only two homozygous mutations (243T>A, S81X) and (IVS26-15A>G) have been reported in this gene in primary microcephaly patients [11]. The gene *CDK5RAP2 *has a genomic size of 191290 bps and contains 34 exons, with a transcript length of 6230 bps and 1893 translated residues [11,27]. The *CDK5RAP2 *gene has predicted to contain one N-terminal spindle associated domain and two potential chromosome segregation ATPase (SMC, structural maintenance of chromosomes) domains, which are known to play a role in the cohesion and condensation of chromosomes during mitosis [28]. The gene product concentrates at spindles poles during prometaphase and metaphase [11,29]. Its localization to the spindle poles of mitotic cells suggests involvement of a centrosomal mechanism during mammalian brain development [11]. *CDK5RAP2 *has a potential role in the inhibition of centrosomal CDK5 during neurogenesis. CDK5 is a promiscuous protein kinase only functioning in the brain with neuron-specific roles in processes including neurogenesis, neural migration and neurodegeneration [29].

In the present study, we report the identification of a previously described nonsense mutation 243 T>A (S81X) in exon 4 of *CDK5RAP2 *gene in a Pakistani family of Kashmiri origin.

## Methods

### Ascertainment of study subjects

**Figure 1:**
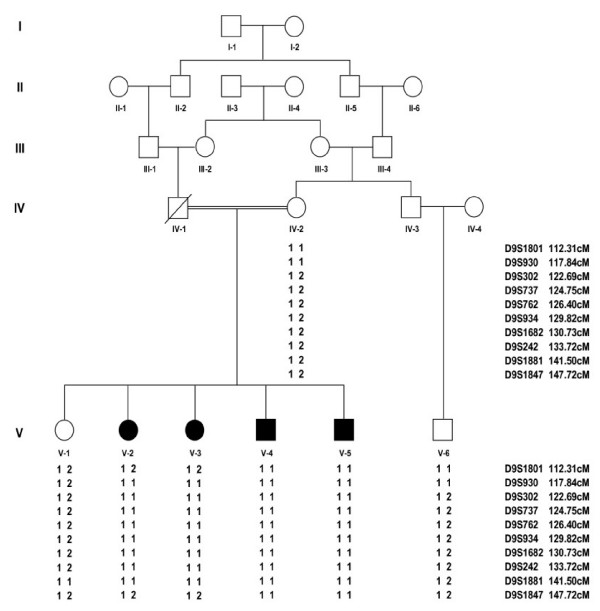
Pedigree drawing of family A. Filled symbols represents individuals with MCPH phenotype, while clear symbols are for unaffected individuals. Haplotypes are shown beneath each genotyped individual. Microsatellite analysis is consistent with linkage of the family to MCPH3.

The study was approved by the Institutional Review Board (IRB) of Quaid-i-Azam University, Islamabad, Pakistan. Informed consent was obtained from all family members who agreed to participate in the study. A Pakistani consanguineous family of Kashmiri origin with four affected individuals showing primary microcephaly was studied. Medical and family history and information on pedigree was obtained from multiple family members. Family pedigree provided convincing evidence of autosomal recessive mode of inheritance (Figure 1) and consanguineous loops accounted for all the affected persons being homozygous for the mutant allele. To exclude the possibility of chromosomal aberrations as the cause of microcephaly, G-banding chromosomal analysis was carried out in one affected individual.

### Extraction of genomic DNA and genotyping

Genomic DNA was extracted from whole blood followed by a standard protocol [30]. To establish linkage of the family to known MCPH loci a minimum of five microsatellite markers from each of the candidate regions were genotyped in all the available individuals. Genetic map distances of the markers used were obtained from Rutgers Combined Linkage-Physical map [31]. Physical positions of the markers were taken from NCBI Build 36.1 of the International Human Genome Sequence Consortium 2007 [32] PEDCHECK [33] was used to identify Mendelian inconsistencies. Two point LOD score was calculated using MLINK program of the FASTLINK computer package [34]. Multipoint LOD score was calculated by ALLEGRO [35]. An autosomal recessive mode of inheritance with complete penetrance and a disease allele frequency of 0.001 were used for the analysis.

PCR for each primer was performed in 25 μl reaction volume containing 40 ng of genomic DNA, 240 nM of primers, 200 uM of each dNTP, I U of Taq DNA polymerase (Fermentas, UK), and 2.5 μl reaction buffer (KCl 50 mM, Tris-HCl pH 8.3, MgCl_2 _15 mM). Details of microsatellite markers used for genotyping and PCR amplification conditions have been described elsewhere [16]. PCR products were resolved on 8% non denaturing polyacrylamide gel and genotypes were assigned by visual inspection.

### Sequencing of *CDK5RAP2*

All coding exons and exon/intron splice junctions of *CDK5RAP2 *gene (Gene Bank ID: NM_18249.4), were PCR amplified from genomic DNA of two affected and one normal individual of the family using primer sequences designed from intronic sequences of the gene. These sequences, designed by using Primer3 software [36], are different from those described previously [11]. Primer sequences used to amplify exons of *CDK5RAP2 *gene and their respective amplification conditions are available on request.

After purification of PCR amplified product with Rapid PCR amplification system (Marligen Bio-sciences, Ijamsville, MD, USA) sequencing was performed with the Big-Dye Terminator version 3.1 Cycle Sequencing Kit, together with an ABI Prism 310 Genetic Analyzer (Applera, Foster City, CA, USA). Sequence variants were identified via Bioedit, sequence alignment editor version 6.0.7. When a potentially functional sequence variant was found, respective exon was sequenced in all other family members for whom DNA was available.

## Results

**Figure 2:**
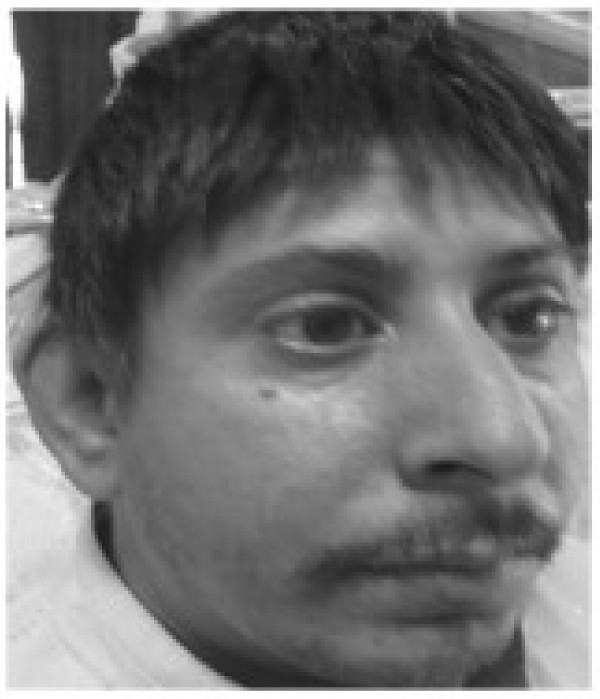
An affected individual (IV-5) of the Pakistani family A. Image has been shown with permission from the respective individual and his parent/guardian.

Clinical history indicated that microcephaly was present by birth in all affected individuals. Head circumferences of affected individuals were 4–7 standard deviations below the age- and sex-related means. Affected individuals examined were 18 to 30 years old and mental retardation ranged from mild to moderate in severity. The facial features of the affected individuals are normal except an indistinct slopping forehead (Figure 2). With the exception of intellectual impairment, no other neurological problems were observed in the affected individuals. The intelligence quotient (IQ) scores for affected individuals, measured at Children Hospital Lahore Pakistan, ranged from 51 to 65. No environment causes could be found to explain the findings of MCPH in this family.

**Figure 3:**
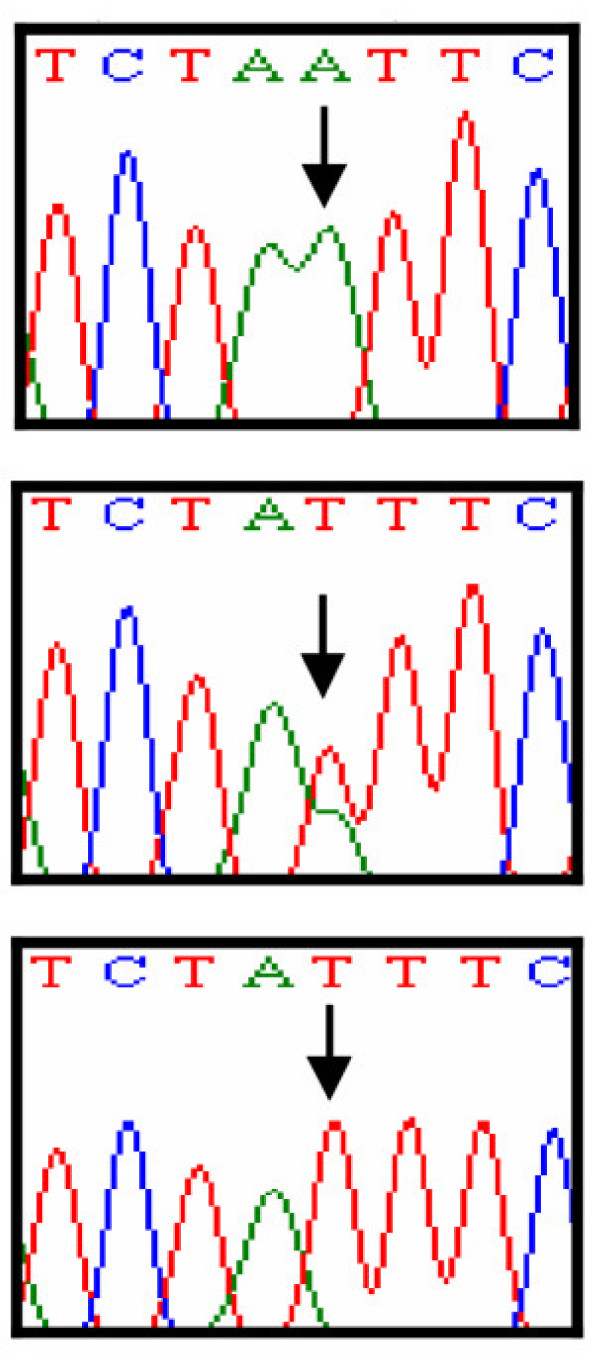
Automated DNA sequence analysis of *CDK5RAP2 *gene mutation in the family. Upper panel represents the sequence in an affected individual, middle panel in heterozygous carrier and lower panel in normal individual of the family.

**Figure 4:**
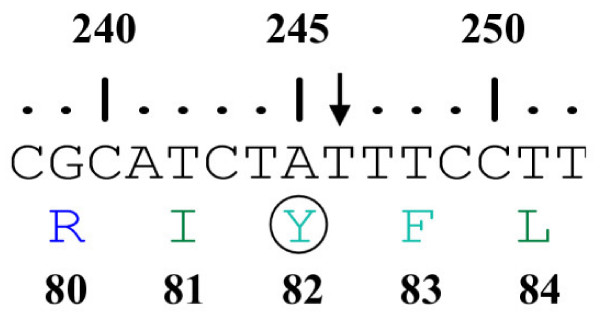
A schematic representation of the nucleotide sequence (238–252) of exon 4 of *CDK5RAP2 *gene. An arrow indicates the nucleotide number 246 which is substituted by A in the patient DNA resulting in the mutation Y82X. The numbers above the nucleotide sequence represent the nucleotide numbers in the gene. The numbers below the single letter code for amino acids represent the amino acid position in the protein.

Linkage of the family to MCPH3 locus was based on the observation that all affected individuals were homozygous with markers in the candidate region of MCPH3 (Figure 1). According to International Human Genome Sequence Consortium 2007 [32], a single 25 Mb region of shared homozygosity- and allele-sharing among the four affected individuals in this family was identified at MCPH3 locus on chromosome 9q31.2–q34.1 between markers D9S1801 and D9S1847. The maximum two point LOD score of 1.58 (θ = 0) was obtained with several markers including D9S302, D9S737, D9S762, D9S934, D9S1682 and D9S242 [Table 1]. The maximum multipoint LOD score of 3.02 was obtained with same markers in the candidate region. Sequence analysis of exon 4 of the gene revealed a T to A transition at nucleotide position 246 in all the affected individuals, resulting in immediate premature stop at amino acid position 82 (Y82X). This mutation was present in heterozygous state in the obligate carriers of the variant (Figure 3, 4). To ensure that the mutation did not represent neutral polymorphisms in the population, a panel of 100 unrelated unaffected individuals (200 chromosomes) was screened for the mutations and it was not identified outside the family.

**Table 1:** Two-point LOD score results between the MCPH3 locus and chromosome 9 markers

			**LOD score at recombination fraction θ =**					
			
**Marker**	Genetic distance*	Physical distance**	**0.00**	**0.01**	**0.05**	**0.1**	**0.2**	**0.3**
D9S1801	112.31	109425991	- inf	-2.73	-1.39	-0.85	-0.37	-0.14
D9S930	117.84	114276019	1.28	1.25	1.14	1.00	0.71	0.43
D9S302	122.69	N.A	1.58	1.54	1.39	1.19	0.82	0.47
D9S737	124.75	118592918	1.58	1.54	1.39	1.19	0.82	0.47
D9S762	126.40	120135547	1.58	1.54	1.39	1.19	0.82	0.47
D9S934	129.82	120135477	1.58	1.54	1.39	1.19	0.82	0.47
D9S1682	130.73	124033005	1.58	1.54	1.39	1.19	0.82	0.47
D9S242	133.72	125908748	1.58	1.54	1.39	1.19	0.82	0.47
D9S1881	141.5	126019246	0.96	0.95	0.89	0.79	0.57	0.33
D9S1847	147.72	134426754	- inf	-2.16	-0.89	-0.45	-0.13	-0.04

*-Cumulative sex-averaged Kosambi genetic map distances (cM) from the Rutgers combined linkage-physical map of the human genome [31].

**-Sequences-based physical map distances in bases according to NCBI Build 36.1 of the human reference sequence [32].

## Discussion

The study presented here described the identification of a recurrent non-sense mutation (Y82X) in *CDK5RAP2 *gene in a consanguineous Pakistani family of Kashmiri origin linked to MCPH3 locus on chromosome 9q31.2–q34.1.

Elsewhere, Bond et al. [11] have reported two homozygous mutations (243T>A and IVS26-15A>G) in *CDK5RAP2 *gene in two families from Pakistan linked to MCPH3 locus. According to the reference sequence (NM_18249.4) the non-sense mutation [(246T>A) Y82X] in *CDK5RAP2 *gene, reported here, is the same as that designated as S82X; [11]. Genomic and cDNA sequence (Gene Bank ID: NM_18249.4) comparison revealed that the actual position of nucleotide conversion is 246T>A, which results in immediate premature stop at amino acid position 82. Also the substituted amino acid was found to be Tyrosine (Y) and not Serine (S) as reported previously [11]. These findings are supported by ENSEMBL genome browser [37] (Gene ID: ENSG00000136861, Transcript ID: E T00000349780, Peptide ID: ENSP00000343818). Alignment of the transcript sequence (NM_18249.4*CDK5RAP2 *transcript variant 1) with the genomic sequence (Genomic ref NC_000009.10 c122382258-122190968 Homo sapiens chromosome 9, reference assembly, complete sequence) of *CDK5RAP2 *gene resulted in 38 hits referring to the presence of 38 translated exons in *CDK5RAP2 *transcript variant 1. ENSEMBL entry corresponds to other database identifiers as well i-e CCDS (ID: 6823.1), Entrez Gene (ID: 55755), UniProtKB/Swiss-Prot [38] (ID: Q96SN8).

Although many ethnic groups speaking different languages such as Kashmiri, Hindko, Shina and Pashto live in Northern Pakistan but according to George Grieson's classification all these ethnic groups are descends of a common ancestor of proto Indo-Aryan origin [39]. Recurrence of Y82X mutation in *CDK5RAP2 *gene in our family of Kashmiri origin may indicate confinement of this rare haplotype within Northern Pakistani population. This family is only the third MCPH family, which showed linkage to MCPH3 locus. Family with primary microcephaly belongs to Rajput Biradiri of District Mirpur, in Azad Jammu and Kashmir, a region bordering India and Pakistan. Due to social and religious customs, consanguineous marriages are a common practice in this population.

MCPH is thought to affect neuronal precursor cell division and hence, primarily a consequence of neurogenic mitosis [1,2]. Immunohistochemistry and confocal microscopy of N-terminal antibodies in HeLa cells showed presence of CDK5RAP2 at centrosome throughout mitosis and it has been hypothesized that an unidentified centrosomal mechanism controls the number of neurons generated by neural precursor cells [11]. Therefore, it may be concluded that mutations in *CDK5RAP2 *affect neurogenic mitosis by reducing the number of microtubules needed to build mitotic spindle and astral microtubule network [29].

## Conclusion

Identification of a recurrent mutation (Y82X) in *CDK5RAP2 *gene in the family studied strengthens the role of this particular mutation in the pathogenesis of the MCPH.

## Competing interests

The author(s) declare that they have no competing interests.

## Authors' contributions

MJH participated in the design of the study, performed PCR, gene sequencing and manuscript writing. MK participated in the design of the study, performed PCR and gene sequencing. ZA, GA and PJ studied family, collected blood samples and extracted DNA. MSC performed sequencing, analyzed the data and participated in manuscript preparation. WA analyzed the data, participated in manuscript preparation and collected funds for the study. All authors read and approved the final manuscript.

## Pre-publication history

The pre-publication history for this paper can be accessed here:


